# Multilocular Thymic Cyst in a Young, Otherwise Healthy Woman: A Case Report

**DOI:** 10.7759/cureus.11210

**Published:** 2020-10-28

**Authors:** Christos Damaskos, Nikolaos Garmpis, Anna Garmpi, Vasiliki E Georgakopoulou, Periklis Tomos

**Affiliations:** 1 Renal Transplantation Unit, Laiko General Hospital, Athens, GRC; 2 Medicine, N.S. Christeas Laboratory of Experimental Surgery and Surgical Research, National and Kapodistrian University of Athens, Athens, GRC; 3 Second Department of Propedeutic Surgery, Laiko General Hospital, Athens, GRC; 4 Medicine, National and Kapodistrian University of Athens, Athens, GRC; 5 First Department of Propedeutic Internal Medicine, Laiko General Hospital, Athens, GRC; 6 Pulmonology Department, Laiko General Hospital, Athens, GRC; 7 First Pulmonology Department, Sismanogleio Hospital, Athens, GRC; 8 Department of Thoracic Surgery, Attikon University Hospital, National and Kapodistrian University of Athens, Athens, GRC

**Keywords:** thymic, cyst, multilocular, mediastinal

## Abstract

Thymic cysts are rare lesions, accounting approximately for 1% of all mediastinal masses. We report a case of a 36-year old woman who presented preoperatively with a calcified mass shadow found on a routine chest radiograph X-ray. After further investigation with chest computed tomography (CT), magnetic resonance imaging (MRI), and tests for Myasthenia gravis, a benign mediastinal cyst was diagnosed and the patient underwent median sternotomy and complete surgical excision of the lesion. The histological examination described a multilocular thymic cyst. Thymic cysts are usually associated with thymic epithelial tumors, such as thymomas, or multisystemic morbid conditions such as human immunodeficiency virus (HIV) infection, rheumatologic disease, and Myasthenia gravis. At all follow-up examinations to date, the patient remains healthy.

## Introduction

Thymic cysts are rare lesions, accounting for approximately 1% of all mediastinal masses. They are classified into two types, namely, unilocular and multilocular, with the latter presenting with a lower incidence [1-3]. The pathogenesis of these two subsets differs significantly. Indeed, unilocular mediastinal cysts are mostly present at birth, whereas multilocular cysts are thought to be developed under the effect of an underlying inflammatory process [4]. Furthermore, the presence of rim calcification in a mediastinal cyst is most likely to be associated with chronic inflammation or a cystic tumor such as cystic teratoma [5].

Histologically, thymic cysts consist of a thick and fibrous wall of epithelium and they include serous fluid or gelatinous content. Inflammation is also evident [2-3].

In general, thymic cysts are asymptomatic and consist of a random finding. Symptoms such as dysphagia, dyspnea, cough, thoracic pain, or Horner syndrome may exist, depending on the size and location of the cyst [1,3,6].

Despite the fact that thymic cysts may be present in any anatomic place between the neck and diaphragm, the majority of them are localized in the anterior mediastinum. Differential diagnosis includes mesothelial and bronchogenic cysts, cystic teratomas or lymphangiomas, and malignant tumors [1,3].

Broadly, thymic cysts are radiologically diagnosed. While they may be presented in chest radiograph X-ray, the chest computed tomography (CT) is the most diagnostic method, as it describes the size, shape, characters, and the relations with adjacent tissues [7].

As there is no consensus on the appropriate therapeutic approach for thymic cysts, surgery seems to be the gold standard, as it decreases the possibility of infections or recurrence [7-8]. Surgical procedures, such as thoracotomy, sternotomy, video-assisted thoracoscopic surgery (VATS), and robot-assisted thoracoscopic surgery (RATS), have been applied [3,9]. When operation cannot be performed, less invasive methods, such as sclerosing with ethanol, have been reported [10].

Herein, we present a case of a young, otherwise healthy woman with an incidental finding on routine chest X-ray that was eventually diagnosed as a multilocular thymic cyst. Notably, clinical and laboratory examinations failed to reveal the presence of either a chronic inflammatory process or any underlying malignancy.

## Case presentation

**Figure 1:**
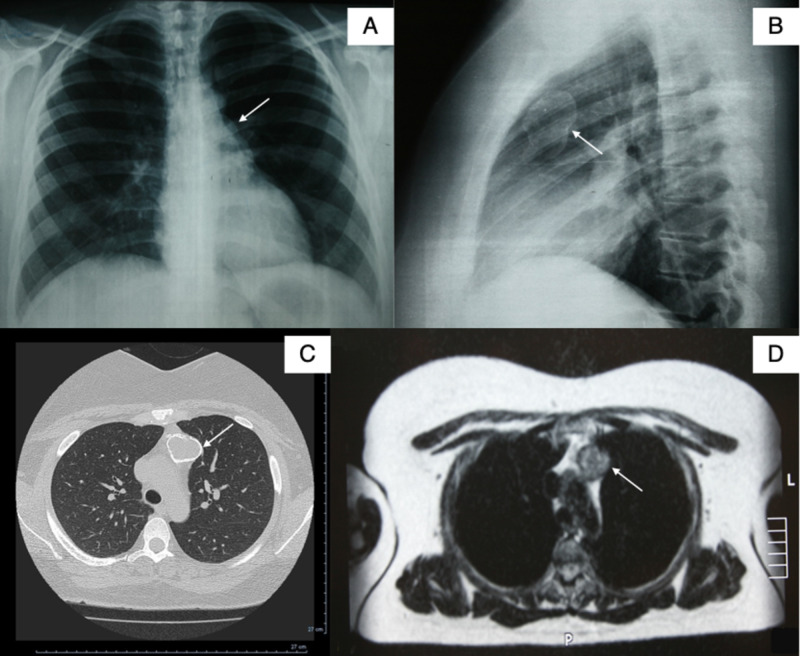
Chest X-ray, computed tomography, and magnetic resonance imaging A, B: Chest radiograph X-ray showing a calcified mass shadow; C: chest computed tomography showing an ellipsoid, well-defined, encapsulated cyst with low attenuation and rim calcification located in the anterior mediastinum; D: Magnetic resonance imaging showing a well-circumscribed cystic lesion with fluid content of low intensity on T1-weighted images and with no adhesion to the surrounding tissues

A 36-year-old woman was referred to our department for further investigation of a calcified mass shadow found on a routine chest radiograph X-ray (Figures 1A-1B). Her physical examination and medical history were unremarkable. Chest computed tomography (CT) revealed an ellipsoid, well-defined, encapsulated cyst with low attenuation and rim calcification located in the anterior mediastinum (Figure 1C). Magnetic resonance imaging (MRI) showed a well-circumscribed cystic lesion with fluid content of low intensity on T1-weighted images and with no adhesion to the surrounding tissues (Figure 1D). Edrophonium test (Tensilon test) and serological test for acetylcholine receptor antibodies and muscle-specific tyrosine kinase antibodies were negative for Myasthenia gravis. Serological testing for HIV infection was negative as well.

**Figure 2:**
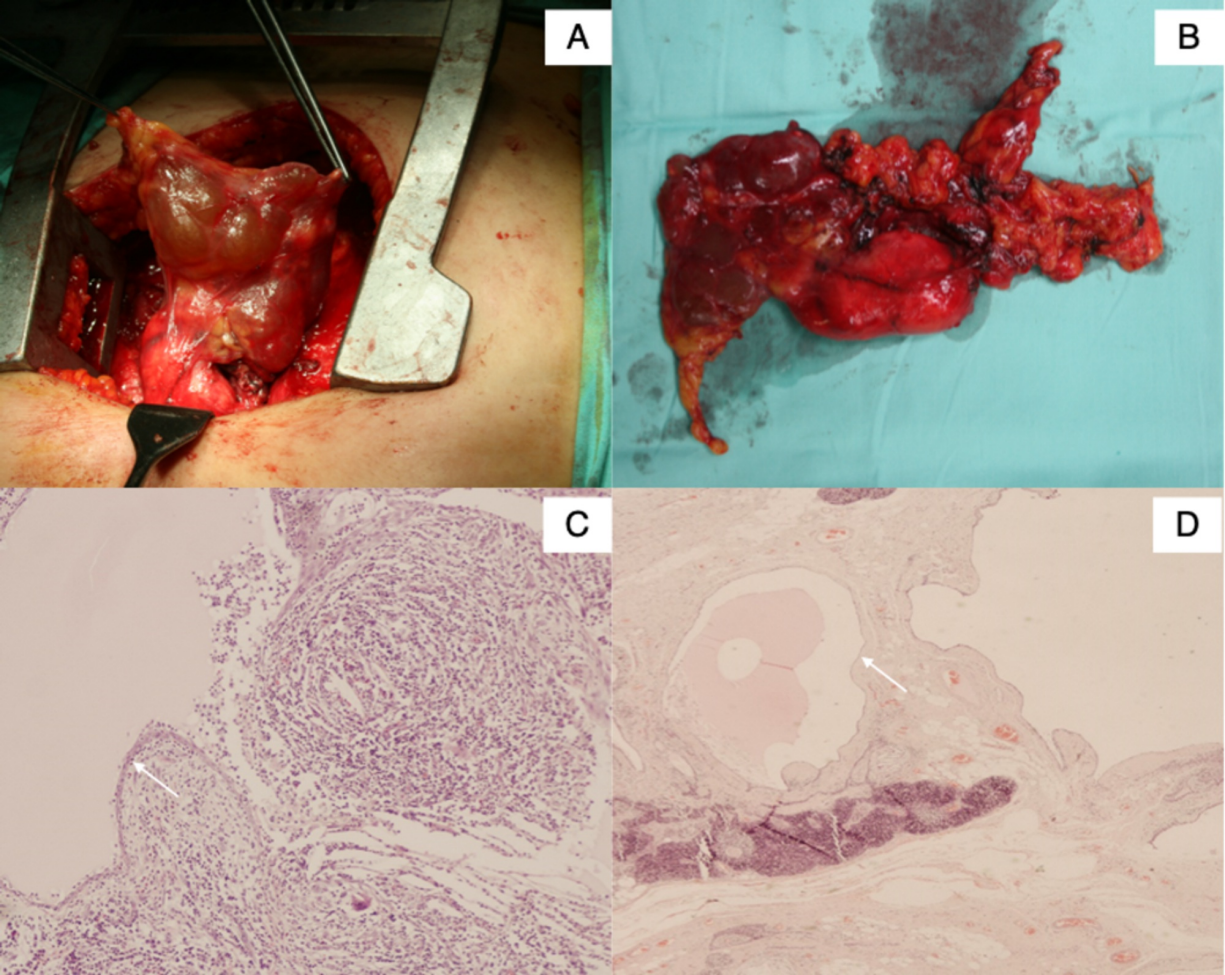
Median sternotomy, surgical specimen of resected encapsulated thymic cyst, and magnification of histologic examination A: Median sternotomy for complete surgical excision of the cystic lesion; B: Surgical specimen of resected encapsulated thymic cyst; C, D: A small and large magnification of the histologic examination reveals the presence of focal calcification as well as focal dense inflammatory infiltrates. The cyst is lined with either single or multiple layers of squamous epithelium where the presence of epithelioid cells and some multinucleated foreign type giant cells are also noted, in accordance with the cholesterol granulomas observed macroscopically. These findings are more consistent with an acquired rather than a congenital thymic cyst.

A benign mediastinal cyst was established as the working diagnosis but due to the observation of a non-accessible lymph node proximal to the left brachiocephalic vein, the patient underwent median sternotomy (Figure 2A) and complete surgical excision of the lesion (Figure 2B). The histological examination described a multilocular cystic lesion with a dense fibrous wall containing calcification and fragments of thymic tissue. The wall of the cyst was heterogeneously lined with single or multiple layers of squamous epithelial cells with foci of epithelial hyperplasia. Cholesterol granuloma formation and inflammatory cells, such as lymphocytes and epithelioid cells, were also observed locally (Figures 2C-2D). The overall histological examination was compatible with the diagnosis of a benign multilocular thymic cyst. Applying the Stanford diagnostic criteria of surgical pathology for the lesion indicates that it is most probably acquired rather than congenital due to the focal areas of hyperplasia as well as the presence of cholesterol granulomata and the observed inflammatory elements. The lymph node had non-specific reactive changes.

## Discussion

Thymic cysts are rare lesions, most often incidentally diagnosed [1]. In general, multilocular thymic cysts are usually associated with thymic epithelial tumors, such as thymomas or underlying multi-systemic morbid conditions, and autoimmune diseases such as HIV infection, rheumatologic diseases, or Myasthenia gravis [11-13]. Although a few cases of pure calcified thymic cysts have been reported in the literature, most cysts with calcification concern cystic teratomas [4]. Besides the presence of a tumor, calcification may be attributed to an underlying chronic inflammatory process. In our case, however, no evidence of pathology was found neither at the time of surgery nor after three years of follow-up.

According to retrospective clinical data, thymic cysts consist of 1%-5% of all mediastinal masses and 5% of anterior mediastinal masses [14-16]. According to a recent study, the median age of diagnosis is 49.5 years [17]. Although generally asymptomatic, thymic cysts have been associated with symptoms such as chest pain, superior vena cava syndrome, wheezing, or dyspnea due to either mass effect or infection. Pathologically, thymic cysts are classified as congenital (in association with a branchial cleft defect) or acquired [18]. The former, lined by single-layered epithelium contain Hassall corpuscles and may be located in both neck and mediastinum [19]. On the other hand, acquired cysts - as in the presented case - contain reactive immune cell populations and are characterized by the presence of cholesterol granulomas in pathology examination [2,18-19].

With regard to the treatment, no consensus exists on the appropriate approach for benign thymic cysts. Surgical removal seems to be the preferred therapeutic strategy, as it decreases the possibility of complications or recurrence [7-8]. Surgical treatment includes thoracotomy, sternotomy, VATS, and RATS [3,9]. In our case, the size of the cyst and a non-accessible lymph node proximal to the left brachiocephalic vein led to the complete surgical excision of the lesion through a median sternotomy.

## Conclusions

In conclusion, an acquired thymic cyst represents a rare entity, usually harboring diseases that need to be excluded, namely, HIV infection, lymphoma, and autoimmune diseases. In our case, however, no such diagnosis was established, raising questions regarding the initiating factors contributing to the disease progress. The lack of broad surgical experience and the association with other mediastinal diseases has also prevented the development of a standardized surgical approach with a level of evidence beyond the expert’s opinion.
